# Exploring the diagnostic value of ultrasound radiomics for neonatal respiratory distress syndrome

**DOI:** 10.1186/s12887-024-04704-3

**Published:** 2024-03-25

**Authors:** Weiru Lin, Junxian Ruan, Zhiyong Liu, Caihong Liu, Jianan Wang, Linjun Chen, Weifeng Zhang, Guorong Lyu

**Affiliations:** 1 Department of Ultrasound, Quanzhou Maternity and Children’s Hospital, No. 700 Fengze Road, Fengze Street, Quanzhou, Fujian Province 362000 China; 2 Department of Neonatal Intensive Care Unit, Quanzhou Maternity and Children’s Hospital, No. 700 Fengze Road, Fengze Street, Quanzhou, Fujian Province 362000 China; 3 Department of Ultrasound, Second Affiliated Hospital of Fujian Medical University, No. 34 North Zhongshan Road, Licheng District, Quanzhou, Fujian Province 362000 China; 4 Quanzhou Medical College, No. 2 Anji Road, Luojiang District, Quanzhou, Fujian Province 362000 China

**Keywords:** Radiomics, Machine learning, Neonatal respiratory distress syndrome, Ultrasound diagnosis, Predictive model

## Abstract

**Background:**

Neonatal respiratory distress syndrome (NRDS) is a prevalent cause of respiratory failure and death among newborns, and prompt diagnosis is imperative. Historically, diagnosis of NRDS relied mostly on typical clinical manifestations, chest X-rays, and CT scans. However, recently, ultrasound has emerged as a valuable and preferred tool for aiding NRDS diagnosis. Nevertheless, evaluating lung ultrasound imagery necessitates rigorous training and may be subject to operator-dependent bias, limiting its widespread use. As a result, it is essential to investigate a new, reliable, and operator-independent diagnostic approach that does not require subjective factors or operator expertise. This article aims to explore the diagnostic potential of ultrasound-based radiomics in differentiating NRDS from other non-NRDS lung disease.

**Methods:**

A total of 150 neonatal lung disease cases were consecutively collected from the department of neonatal intensive care unit of the Quanzhou Maternity and Children’s Hospital, Fujian Province, from September 2021 to October 2022. Of these patients, 60 were diagnosed with NRDS, whereas 30 were diagnosed with neonatal pneumonia, meconium aspiration syndrome (MAS), and transient tachypnea (TTN). Two ultrasound images with characteristic manifestations of each lung disease were acquired and divided into training (*n* = 120) and validation cohorts (*n* = 30) based on the examination date using an 8:2 ratio. The imaging texture features were extracted using PyRadiomics and, after the screening, machine learning models such as random forest (RF), logistic regression (LR), K-nearest neighbors (KNN), support vector machine (SVM), and multilayer perceptron (MLP) were developed to construct an imaging-based diagnostic model. The diagnostic efficacy of each model was analyzed. Lastly, we randomly selected 282 lung ultrasound images and evaluated the diagnostic efficacy disparities between the optimal model and doctors across differing levels of expertise.

**Results:**

Twenty-two imaging-based features with the highest weights were selected to construct a predictive model for neonatal respiratory distress syndrome. All models exhibited favorable diagnostic performances. Analysis of the Youden index demonstrated that the RF model had the highest score in both the training (0.99) and validation (0.90) cohorts. Additionally, the calibration curve indicated that the RF model had the best calibration (*P* = 0.98). When compared to the diagnostic performance of experienced and junior physicians, the RF model had an area under the curve (AUC) of 0.99; however, the values for experienced and junior physicians were 0.98 and 0.85, respectively. The difference in diagnostic efficacy between the RF model and experienced physicians was not statistically significant (*P* = 0.24), whereas that between the RF model and junior physicians was statistically significant (*P* < 0.0001).

**Conclusion:**

The RF model exhibited excellent diagnostic performance in the analysis of texture features based on ultrasound radiomics for diagnosing NRDS.

## Background

Neonatal respiratory distress syndrome (NRDS) is a condition in which newborns experience respiratory distress shortly after birth, primarily due to progressive alveolar atrophy caused by a deficiency in alveolar surface type II active substances [1]. Traditionally, the diagnosis of NRDS is based on clinical manifestations and radiographic examinations [2]. Previously, the diagnosis of lung disease was considered inappropriate for ultrasonography [3, 4]. Recent studies have shown that lung ultrasound has good diagnostic sensitivity and specificity for various lung diseases in neonates and children [5–7]. However, an accurate ultrasound diagnosis of pulmonary diseases requires systematic operator training for operation and diagnosis. Moreover, this often relies on the subjective judgment of the operator, which may delay the diagnosis and treatment of the child if the judgment is incorrect. Therefore, finding and establishing a more objective and reliable diagnostic method for NRDS is important for clinical ultrasonography.

Radiomics is a novel and non-invasive technique that can extract massive amounts of feature data from images that are difficult to discern using human vision. It can achieve a quantitative representation of image features such as grayscale, texture, and morphology [8]. Machine learning algorithms can be utilized to analyze data accurately, establish predictive models, reduce subjective judgments, and provide objective quantitative predictive data to assist physicians’ decisions [9, 10]. By analyzing lung ultrasound images of patients with coronavirus disease (COVID-19), some scholars [11] found that the support vector machine (SVM) model demonstrated better accuracy in assessing the severity of pleural line changes, which is significant for accurately assessing patients’ diseases. A recent study [12] shows that integrating seven machine learning models selected for the prediction of preoperative 2-deoxy-2-[fluorine-18]fluoro-D-glucose (^[18 F]^ FDG) positron emission tomography/computed tomography (PET/CT) radiographic features to predict the pathological aggressiveness of lung cancer had the highest diagnostic efficacy and better stability. These studies demonstrated the application of histological imaging in lung diseases.

Ultrasound imaging is not commonly used to diagnose pulmonary diseases. This study aimed to investigate the diagnostic efficacy of ultrasound-assisted diagnosis of NRDS.

## Methods

### Patients and data collection

A total of 150 inpatients who underwent lung ultrasound examination at the neonatal intensive care unit of Quanzhou Maternity and Children’s Hospital between September 2021 and November 2022 were included in this study. The patients were divided into training (*n* = 120) and verification cohorts (*n* = 30) based on the time of admission in an 8:2 ratio. The diagnostic criteria for NRDS were based on the European Consensus Guidelines on the Management of Respiratory Distress Syndrome:2022 Update. These criteria include (1) clinical manifestations such as shallow breathing, dyspnea, and expiratory moans appearing immediately after birth or within 4–6 h, which gradually worsen with time, along with blue and gray complexion, three concave signs, nasal flapping, and progressive cyanosis that does not improve with oxygen. Additionally, the pulmonary breath sounds and audible crackles decreased at the end of deep inspiration; (2) blood gas analysis indicated hypoxemia, increased blood carbon dioxide, metabolic acidosis, and respiratory acidosis; (3) chest X-ray findings showed a diffuse decrease in the transmittance of both lungs, ground-glass opacities in mild cases of both lung fields, the bronchial inflated phase as the disease progressed, and white lung formation in severe cases.

**Fig. 1:**
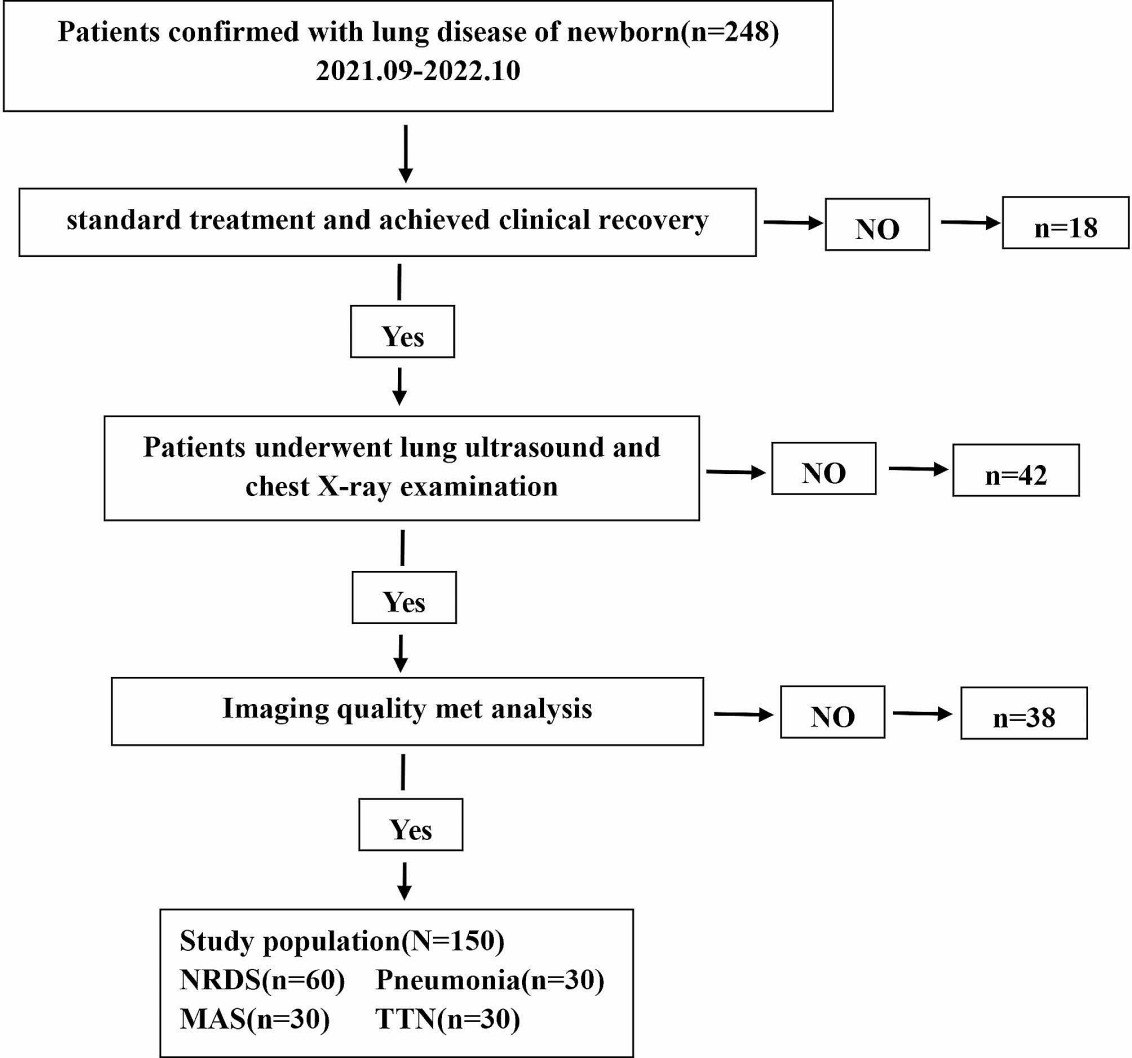
The flowchart outlining the inclusion and exclusion criteria for study subjects. NRDS = neonatal respiratory distress syndrome, MAS = meconium aspiration syndrome, TTN = transient tachypnea of the newborn

The exclusion criteria for this study were as follows: (1) Congenital developmental abnormalities such as congenital pulmonary dysplasia, thoracic malformation, posterior nasal atresia, congenital diaphragmatic hernia, and severe congenital heart disease; (2) Restrictive lung ventilation diseases, including severe pneumothorax and severe abdominal distention; (3) Prophylactic use of alveolar surfactant after birth; (4) Diagnosis of pulmonary cyst adenomatous malformations during both fetal and postpartum periods. The procedures for incorporating and excluding study participants are depicted in Fig. 1.

### Ultrasound image acquisition

A GE LOGIQ P6 color Doppler ultrasound was utilized in this study, and a line array probe with a frequency of 9–12 MHz was selected. Only one focal point was selected and aligned with the pleural line. The harmonics were turned off, and the sweep depth was set to 3–4 cm. Two doctors with extensive experience in lung ultrasound diagnosis adjusted and optimized the image quality to capture the optimal images. Two ultrasound images of each patient were obtained and saved in digital imaging and communications in medicine (DICOM) format.

### Lesion segmentation and radiomic feature extraction

The workflow for imaging histology involves several steps, including regions of interest (ROI) cutting, feature selection, feature extraction, and model construction. All ultrasound images meeting the inclusion criteria were obtained using an ultrasound instrument. Two senior physicians specializing in neonatal lung ultrasound manually outlined the ROI of the lesion area using ITK-SNAP 3.8.0 software (http://www.itksnap.org). In cases where images with combined pleural effusion were encountered, pleural effusion was excluded to avoid any potential interference.

### Feature selection and radiomics model construction

**Fig. 2:**
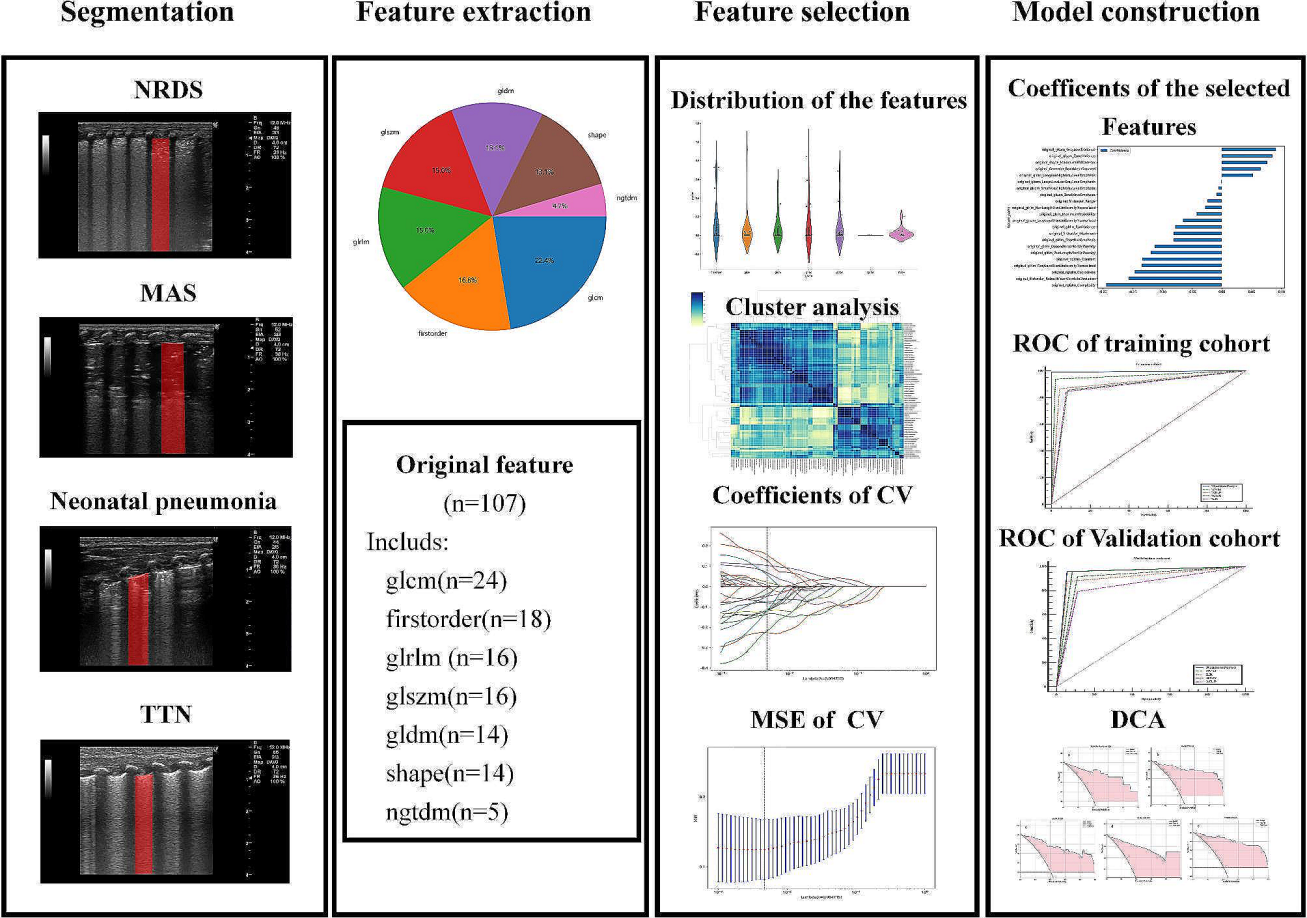
The workflow of the radiomics model construction. In the segmentation section, we delineated the regions of interest (ROI) for lung ultrasound in patients with neonatal respiratory distress syndrome (NRDS), neonatal pneumonia, meconium aspiration syndrome (MAS), and transient tachypnea of the newborn (TTN), respectively. In the feature extraction part, we presented the results of feature extraction and the distribution proportions. In the feature selection section, we showcased the p-value distribution of the extracted features, cluster analysis, the coefficient convergence of the least absolute shrinkage and selection operator (LASSO) regression applied to the features, and the results of ten-fold cross-validation to obtain the optimal λ. In the model construction section, we displayed the features with none zero obtained after feature selection, ROC curves for the training and validation sets, and the decision curve analysis (DCA) for different models

The PyRadiomics module of Python software was used to extract the imaging histology features of ROI. A total of 107 initial imaging histology features were extracted, and after applying a significance threshold of *P* < 0.05, 83 features were retained for further analysis. Any two features with a correlation coefficient greater than 0.9 were identified using Spearman rank correlation coefficient calculation and reduced to a single feature to eliminate redundant features with high repeatability. Furthermore, a greedy recursive strategy was employed to filter irrelevant features, and the remaining features were used to construct a dataset for the least absolute shrinkage and selection operator (LASSO) regression model. Ten-fold cross-validation was performed to obtain the optimal *λ*, and the features with none zero were retained for the prediction model construction (Fig. 2).

### Statistical analysis

All statistical analyses were performed using the SPSS software (version 26.0). For normally distributed measures, descriptive statistics are presented as mean ± standard deviation (±S). A t-test was used for two independent sample groups. Non-normally distributed measures were described using the median and interquartile range, represented as *M* (P25, P75), and the Mann-Whitney U test was used to compare the differences between the groups. Count data were presented as cases and composition ratios (%), and differences between groups were assessed using the chi-square, continuous corrected chi-square, or Fisher’s exact test. The variability of the performance data obtained from the internal validation of each model was analyzed using the McNemar test. Calibration curves were constructed for each model to assess the degree of calibration. Statistical significance was set at *p* < 0.05.

## Results

### Clinical characteristics of patients

The fundamental clinical characteristics of the children in the training and validation cohorts are comparable, as shown in Table 1.

**Table 1:** Baseline characteristics of patients in the training and test cohorts

Characteristics		Training cohort(*n* = 120, %)	Test cohort(*n* = 30, %)	χ^2^ /Z	P
Age, day [M(P25, P75)]	263(216, 277)		264(228, 277)	-0.634	0.526
Weight, g [M(P25, P75)]	2860(1500, 3565)		2925(1950, 3413)	-0.115	0.908
Male	86(71.7)		19(63.3)	0.794	0.373
Eutocia	62(51.6)		16(53.3)	0.027	0.870
History of intrauterine distress	16(13.3)		0(0)	3.188	0.870
History of asphyxia	30(25)		5(16.7)	0.932	0.074
Gestational diabetes mellitus	27(22.5)		7(23.3)	0.010	0.334
Pregnancy-induced hypertension	5(4)		1(3)	0.010	0.922
History of glucocorticoid use	35(29.2)		7(23.3)	0.405	0.755

### Feature extraction and selection for ultrasound images

**Fig. 3:**
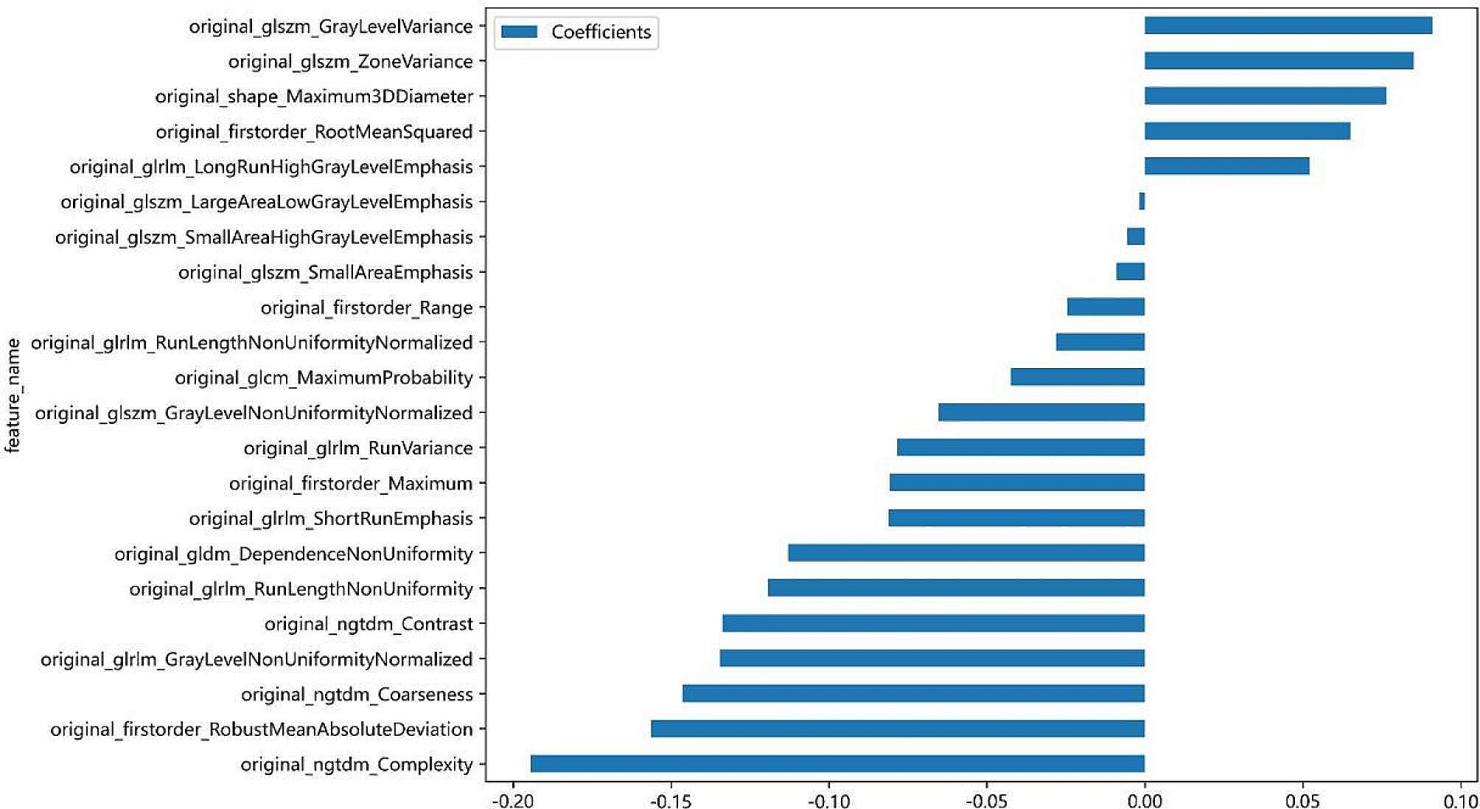
The histogram of the coefficients of the selected features. 22 features that coefficient value was none zero remained, signature was built according to the coefficient value of the selected features

A total of 107 initial image histology features were extracted. Following feature screening by the LASSO algorithm, 22 features with none zero were ultimately incorporated into building the prediction model (Fig. 3).

### Comparison of diagnostic effectiveness of different models

The 22 features with none zero were included in five models: random forest (RF), support vector machine (SVM), multilayer perceptron (MLP), k-nearest neighbor (KNN), and logistic regression (LR). The results demonstrated that all models had high diagnostic efficacy, and no statistically significant differences were observed between the pair-wise comparisons of sensitivity, specificity, positive predictive value, or negative predictive value among the models (*P* > 0.05). In the training cohort, the RF and SVM models showed higher Youden indices, whereas the KNN and LR models showed lower Youden indices (Table 2). In the validation cohort, the RF and SVM models showed higher Youden indices; however, the KNN and MLP models showed lower values. The study found that the RF model exhibited the highest diagnostic efficacy (Table 3).

**Table 2:** Comparison of the diagnostic performance of different models in the training cohort

Model	Sen	Spe	Accuracy	PPV	NPV	AUC	Youden index
RF	98.96%	100%	99.58%	100%	99.31%	0.995	0.9896
SVM	93.75%	97.92%	96.25%	96.77%	95.92%	0.958	0.9167
MLP	86.46%	95.83%	92.08%	93.26%	97.87%	0.911	0.8229
KNN	85.42%	91.67%	89.17%	87.23%	90.41%	0.885	0.7709
LR	84.40%	92.36%	89.17%	88.04%	89.86%	0.884	0.7676

**Table 3:** Comparison of the diagnostic performance of different models in the validation cohort

Model	Sen	Spe	Accuracy	PPV	NPV	AUC	Youden index
RF	95.83%	94.44%	95.00%	92.00%	97.14%	0.951	0.9027
SVM	95.83%	91.67%	93.33%	88.46%	97.06%	0.938	0.8750
LR	87.50%	94.44%	91.67%	91.30%	91.89%	0.910	0.8194
KNN	91.67%	88.89%	90.00%	84.62%	94.12%	0.903	0.8056
MLP	79.17%	88.89%	85.00%	82.61%	86.48%	0.840	0.6806

### Calibration curve comparison

**Fig. 4:**
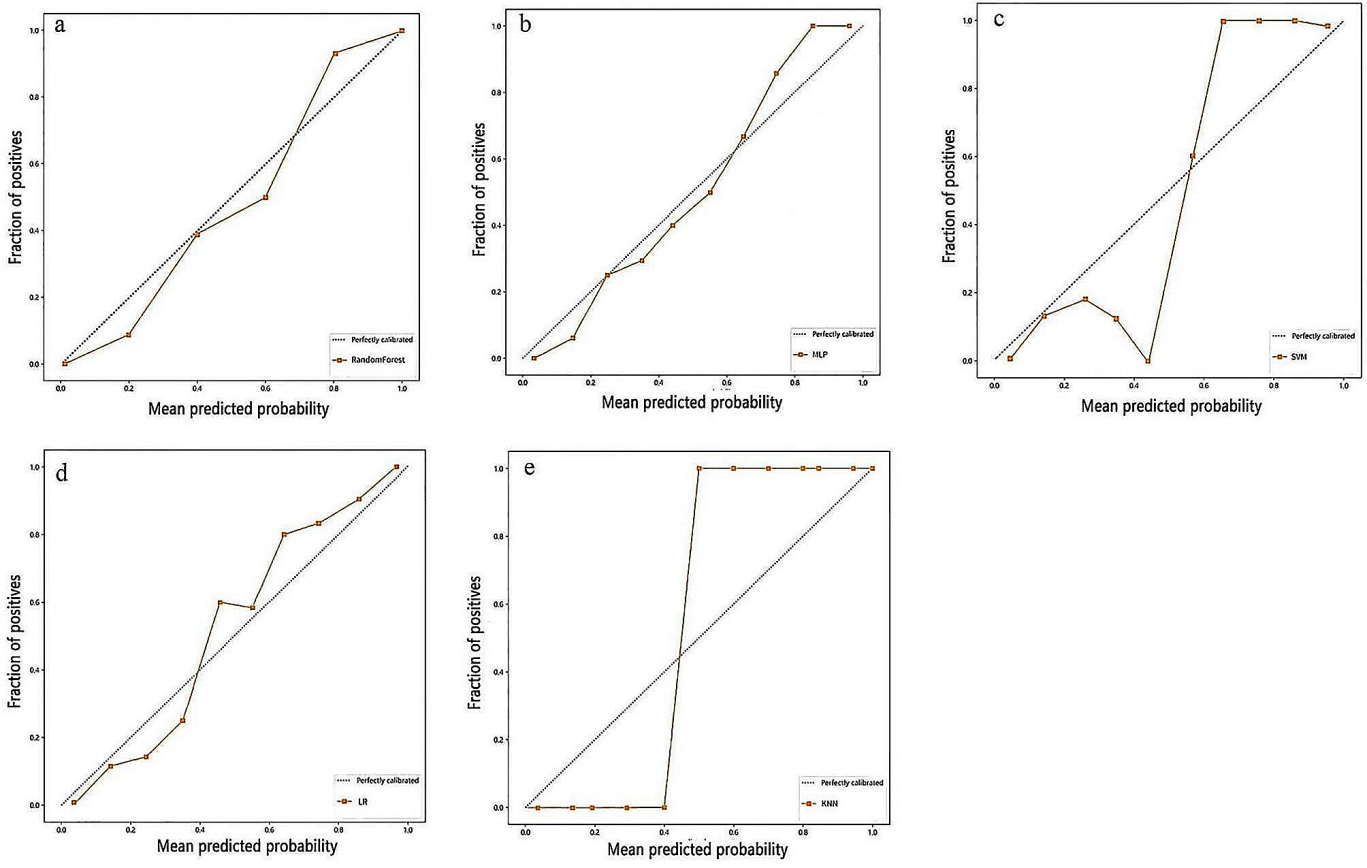
Calibration curves of different models. a$\sim$e: RF、MLP、SVM、LR、KNN model, respectively. The x-axis represents the mean predicted probability, and the y-axis represents the proportion of true positives. The diagonal dashed line represents the reference line of perfect calibration. A model with good calibration will have its calibration curve closer to the reference line. A Hosmer-Lemeshow test with a p-value > 0.05 indicates good model fit

The Hosmer–Lemeshow test showed that the KNN model was poorly calibrated (*p* = 0.004, *p* < 0.05), whereas the RF (*p* = 0.982), MLP (*p* = 0.599), SVM (*p* = 0.462), and LR (*p* = 0.340) models were better calibrated (Fig. 4).

### Comparison of decision curves for different models

**Fig. 5:**
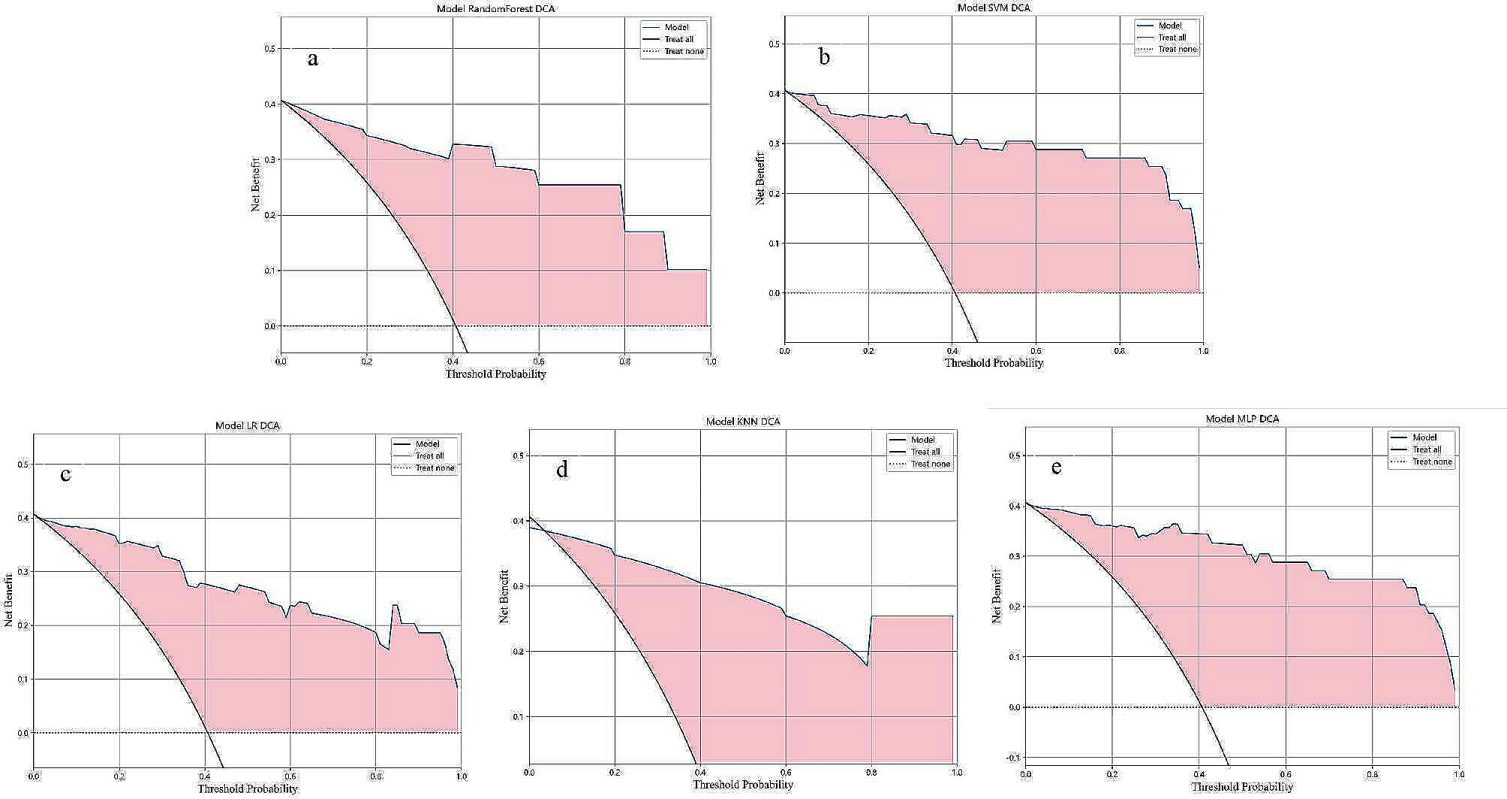
Decision curves of different models. a$\sim$e: RF、SVM、LR、KNN、MLP model, respectively. The horizontal axis represents the threshold probability, while the vertical axis represents the net benefit rate. The larger the red shaded area, the wider the threshold range, and the greater the clinical value of the model

In most cases, diverse models have demonstrated the potential to offer a notable rate of clinical benefit (Fig. 5).

### The diagnostic accuracy of randomly selected lung ultrasound images varies across doctors of differing levels of expertise

The diagnostic efficacy of the RF model and that of the senior physicians (over 5 years of dedicated experience in neonatal lung ultrasound) did not differ significantly regarding AUC, sensitivity, specificity, positive predictive value, or negative predictive value, and both were significantly better than those of junior physicians (3 $\sim$ 5 years of involvement in neonatal lung ultrasound work) (Table 4). Moreover, physicians at different levels exhibited discrepancies in their subjective diagnoses, with mild NRDS and transient respiratory tachypnea in neonates being frequently misdiagnosed.

**Table 4:** Comparison of diagnostic efficacy between different levels of doctors and RF model

Group	Sen	Spe	Accuracy	PPV	NPV	AUC	Youden index
Senior doctor	99.11%^a^	98.82%^a^	98.94%	98.23%^a^	99.41%^a^	0.990	0.9793
RF	98.21%^a^	97.65%^a^	97.87%	96.49%^a^	98.81%^a^	0.979	0.9586
Junior doctor	81.25%	89.41%	86.17%	83.49%	87.86%	0.853	0.7066

^a^: ^a^*P*<0.05 compared with junior doctors

## Discussion

Radiomics, a branch of artificial intelligence, has recently gained increasing attention in clinical medicine because of its ability to extract significant feature data from ultrasound images, representing quantitative information on image features, such as grayscale, texture, and morphology. The integration of machine learning algorithms allows for objective analysis of this data, minimizing subjective judgments and providing physicians with quantitative data to inform their decision-making [9, 13, 14]. Although research on machine learning for lung ultrasound is scarce, both domestically and internationally, scholars such as Cristiana Baloescu et al. [15] have successfully used convolutional neural networks to develop automated detection models for lung ultrasound B-lines, which were effective in assessing the severity of the alveolar interstitial syndrome. Another recent study [16] established a deep learning model to distinguish seven pivotal lung ultrasound features in neonates, demonstrating a commendable average accuracy. However, this model cannot distinguish NRDS from other lung diseases. Notably, limited research exists on imaging histology for identifying NRDS and other neonatal lung diseases using lung ultrasound.

In this study, lung ultrasound images of 60 children with NRDS and 90 children with other lung diseases were analyzed using ultrasound imaging histology. The results indicated that the models performed well in the training and validation cohorts. Specifically, in the training cohort, no statistically significant differences were observed in sensitivity, specificity, positive predictive value, or negative predictive value between the models. However, in the validation cohort, the Jordans were higher for the RF and SVM models and lower for the KNN and MLP models; the KNN model was poorly calibrated, whereas the other models were well calibrated, with the RF model being the best and the MLP and SVM models being the second best. When comparing the diagnostic efficacy of randomly selected lung ultrasound images between different levels of physicians, the RF model showed comparable diagnostic efficacy to senior physicians, with no statistically significant differences in sensitivity, specificity, positive predictive value, or negative predictive value. However, statistically significant differences were observed with junior physicians, and the Youden index was slightly lower than that of senior physicians and significantly higher than that of junior physicians. These results suggest that the RF model has better clinical application value. Furthermore, the RF model demonstrated stable and high application ability and proved to be the optimal model in the study of imaging-based histology-assisted diagnosis of NRDS.

Noteworthy, the RF model exhibits high accuracy and strong model generalization ability owing to the integrated algorithm that incorporates decision-tree-based stochastic attributes. The RF model demonstrated widespread applicability in various scenarios. For instance, Ren et al. [17] showed that the screening and predictive modeling of endometriosis-causing genes based on the RF model exhibited good clinical ability. Moreover, studies by Kwak et al. [18] and C. Venkata Narasimhulu [19] showed that the diagnostic efficacy of RF models is superior to that of experienced sonographers in diagnosing benign and malignant thyroid nodules and classifying benign and malignant renal cancers after noise reduction processing of the images. These studies indicate that the RF model is a useful tool for clinical practice and medical image classification, which is consistent with the RF model selected in this study for differentiating NRDS from non-NRDS lung ultrasound diseases.

This study had several limitations. First, this study used only one instrument model for image collection, and further exploration is required to determine whether the findings can be consistently reproduced using different instrument models. Second, this study was limited to a single-center setting; multicenter studies are needed to verify whether the diagnostic efficacy of the RF model is consistent across different settings. Finally, the sample size of this study was small, and obtaining a larger sample size for analysis would help verify the fundamental clinical characteristics of the children [20] and the stability of the model. These limitations highlight the need for further research to address these issues and improve the robustness of our findings.

## Conclusions

In conclusion, the results of this study indicate that imaging histology analysis based on lung ultrasound images using the RF model resulted in superior diagnostic efficacy compared to other models, as demonstrated by its consistently high performance in both the training and validation cohorts, as well as in the evaluation of calibration curves. These findings suggest that the RF model is a promising approach for diagnosing neonatal respiratory distress syndrome based on lung ultrasound.

## Data Availability

The datasets generated and analyzed during the current study are not publicly due to privacy restrictions but available from the corresponding author upon reasonable request.

## Abbreviations

RF: random forest
SVM: support vector machine
MLP: multilayer perceptron
KNN: k-nearest neighbor
LR: logistic regression
Sen: sensitivity
Spe: specificity
PPV: positive predictive value
NPV: negative predictive value
AUC: area under the curve
MAS: meconium aspiration syndrome
TTN: transient tachypnea
CV: cross validation
MSE: mean square error
ROC: receiver operating characteristic
DCA: decision curve analysis
glcm: grayscale co-occurrence matrix features
first order: first-order features
glrlm: grayscale run-length matrix features
glszm: grayscale size-zone matrix features
gldm: grayscale dependence matrix features
shape: shape feature
ngtdm: neighboring gray tone difference matrix

## References

[1] Sweet D, Carnielli V, Greisen G, Hallman M, Klebermass-Schrehof K, Ozek E, Te Pas A, Plavka R, Roehr C, Saugstad O (2023). European Consensus guidelines on the management of respiratory distress syndrome: 2022 update. Neonatology.

[2] Ismail R, El Raggal N, Hegazy L, Sakr H, Eldafrawy O, Farid Y. Lung Ultrasound Role in diagnosis of neonatal respiratory disorders: a prospective cross-sectional study. Child (Basel Switzerland) 2023, 10(1). 10.3390/children10010173 PMC9857438 36670723

[3] Lichtenstein D (2010). Should lung ultrasonography be more widely used in the assessment of acute respiratory disease?. Expert Rev Respir Med.

[4] Gargani L (2011). Lung ultrasound: a new tool for the cardiologist. Cardiovasc Ultrasound.

[5] Guo BB, Pang L, Yang B, Zhang C, Chen XY, Ouyang JB, Wu CJ (2022). Lung ultrasound for the diagnosis and management of neonatal respiratory distress syndrome: a Minireview. Front Pead.

[6] Liang Z, Meng Q, You C, Wu B, Li X, Wu Q (2021). Roles of lung ultrasound score in the Extubation failure from mechanical ventilation among premature infants with neonatal respiratory distress syndrome. Front Pead.

[7] Jiang Q, Shi L, Shen L, Li X, Huang R, Chen L, Li J, Lyu G (2022). Application value of a New Lung Ultrasound Scoring Method in neonatal respiratory distress syndrome treatment. Ultrasound Med Biol.

[8] Chen Q, Xia T, Zhang M, Xia N, Liu J, Yang Y (2021). Radiomics in Stroke Neuroimaging: techniques, applications, and challenges. Aging Disease.

[9] Hu HT, Wang Z, Huang XW, Chen SL, Kuang M. Ultrasound-based radiomics score: a potential biomarker for the prediction of microvascular invasion in hepatocellular carcinoma. Eur Radiol 2018. 10.1007/s00330-018-5797-0 30421015

[10] Choy G. Khalilzadeh, Omid, Michalski, Mark, Synho, Samir, Anthony E, Pianykh: Current Applications and Future Impact of Machine Learning in Radiology. *Radiology* 2018. 10.1148/radiol.2018171820 PMC6542626 29944078

[11] Carrer L, Donini E, Marinelli D, Zanetti M, Mento F, Torri E, Smargiassi A, Inchingolo R, Soldati G, Demi L (2020). Automatic Pleural line extraction and COVID-19 Scoring from Lung Ultrasound Data. IEEE Trans Ultrason Ferroelectr Freq Control.

[12] Onozato Y, Iwata T, Uematsu Y, Shimizu D, Yamamoto T, Matsui Y, Ogawa K, Kuyama J, Sakairi Y, Kawakami E (2023). Predicting pathological highly invasive lung cancer from preoperative [F]FDG PET/CT with multiple machine learning models. Eur J Nucl Med Mol Imaging.

[13] Lambin P, Rios-Velazquez E, Leijenaar R, Carvalho S, Aerts HJWL (2012). Radiomics: extracting more information from medical images using advanced feature analysis. Eur J Cancer.

[14] Weigel MT, Dowsett M (2010). Current and emerging biomarkers in breast cancer: prognosis and prediction. Endocr Relat Cancer.

[15] Baloescu C, Toporek G, Kim S, McNamara K, Liu R, Shaw M, McNamara R, Raju B, Moore C (2020). Automated Lung Ultrasound B-Line Assessment using a deep learning algorithm. IEEE Trans Ultrason Ferroelectr Freq Control.

[16] Bassiouny R, Mohamed A, Umapathy K, Khan N (2021). An interpretable object detection-based Model for the diagnosis of neonatal lung diseases using Ultrasound images. Annual Int Conf IEEE Eng Med Biology Soc IEEE Eng Med Biology Soc Annual Int Conf.

[17] She J, Su D, Diao R, Wang L (2022). A joint model of Random Forest and Artificial Neural Network for the diagnosis of endometriosis. Front Genet.

[18] Zhang B, Tian J, Pei S, Chen Y, He X, Dong Y, Zhang L, Mo X, Huang W, Cong S (2019). Machine learning-assisted system for thyroid nodule diagnosis. Thyroid: Official J Am Thyroid Association.

[19] Narasimhulu CV. An automatic feature selection and classification framework for analyzing ultrasound kidney images using dragonfly algorithm and random forest classifier. IET Image Proc. 2021;15(9):–.

[20] Amrhein V, Greenland S, McShane B (2019). Scientists rise up against statistical significance. Nature.

